# Influence of context-sensitive rules on the formation of orthographic representations in Spanish dyslexic children

**DOI:** 10.3389/fpsyg.2014.01409

**Published:** 2014-12-04

**Authors:** Paz Suárez-Coalla, Rrezarta Avdyli, Fernando Cuetos

**Affiliations:** Department of Psychology, University of OviedoOviedo, Spain

**Keywords:** dyslexia, orthographic representations, fluency, transparent orthography, context-sensitive rules

## Abstract

Spanish-speaking developmental dyslexics are mainly characterized by poor reading fluency. One reason for this lack of fluency could be a difficulty in creating and accessing lexical representations, because, as the self-teaching theory suggest, it is necessary to develop orthographic representations to use direct reading (Share, 1995). It is possible that this difficulty to acquire orthographic representations can be specifically related to words that contain context-sensitive graphemes, since it has been demonstrated that reading is affected by this kind of graphemes (Barca et al., 2007). In order to test this possibility we compared a group of dyslexic children with a group of normal readers (9–13 years), in a task of repeated reading. Pseudo-words (half short and half long) with simple and contextual dependent rules were used. The length effect reduction on the reading speed, after repeated exposure, was considered an indicator of orthographic representation development, as the length effect is strong when reading unknown words, but absent when reading familiar words. The results show that dyslexic children have difficulties in developing orthographic representations, not only with context-sensitive graphemes, but also with simple graphemes. In contrast to the control children, in the dyslexic group differences between reading times for short and long stimuli remained without significant changes after six presentations. Besides, this happened with sensitive context rules and also with simple grapheme–phoneme conversion rules. On the other hand, response and articulation times were greatly affected by length in dyslexic children, indicating the use of serial reading. Results suggest that the problems related to storing orthographic representations could be caused by a learning deficit, independently of whether the word contained context-sensitive rules or not.

## INTRODUCTION

Dyslexic children learning to read in transparent orthographic systems make relatively few errors in the reading of words when compared with dyslexics using opaque orthographic systems (Rack et al., 1992; Yap and Van der Leij, 1993; Sprenger-Charolles et al., 2000; De Jong and van der Leij, 2002; Nikolopoulos et al., 2003). Several cross-linguistic studies have shown that orthographic depth largely determines the reading skills of dyslexics, so that decoding problems are more evident in opaque orthographic systems, such as English, than in transparent orthographic systems (Wimmer and Goswami, 1994; Landerl et al., 1997). It appears that the high consistency between graphemes and phonemes facilitates learning of the alphabetic code, and consequently reading accuracy, even in dyslexic children.

Dyslexics in transparent orthographic systems, however, fail to achieve an acceptable level of reading speed (Wimmer, 1993; Spinelli et al., 2005); their reading is generally slow and laborious, similar to the reading of dyslexics in deep orthographic systems. (Oney and Goldman, 1984; Landerl, 2001; Zoccolotti et al., 2005; Suárez-Coalla and Cuetos, 2012). As the reading speed problems are more striking than the accuracy problems for these dyslexics (although they are also more error-prone than age-matched children), difficulty in acquiring reading speed is considered a marker of dyslexia in transparent orthographic systems such as Spanish, German, Italian, or Greek (Ziegler et al., 2003; Davies et al., 2007; Constantinidou and Stainthorp, 2009; Wimmer et al., 2010). In particular, dyslexics are much slower than normal children reading long words and non-words (Davies et al., 2007; De Luca et al., 2008; Suárez-Coalla and Cuetos, 2012).

Why don’t dyslexic children develop reading fluency? Is it because they have difficulties learning and automating the grapheme–phoneme conversion rules? Probably, because differences between dyslexic and normal children are bigger in reading non-words (Yap and Van der Leij, 1993; Snowling, 1995), which indicate problems in using the sublexical route. Although in recent years, several authors have questioned this conclusion mostly based on methodological considerations (see Van den Broeck and Geudens, 2012, for a very thorough discussion). But dyslexics are also poorer in reading familiar words (Hatcher et al., 2002), which indicates difficulties to develop orthographic representations of the words. Then, what does prevent them from forming and accessing orthographic representations? According to the self-teaching theory, orthographic representations are developed through accurate and repeated reading (Reitsma, 1989; Share, 1995, 1999; Cunningham et al., 2002; De Jong et al., 2009). If dyslexics have difficulty developing orthographic representations, they should be even slower than normal individuals when reading frequent words, because they also have to read them by the sublexical route; indeed, some studies have confirmed this observation (Defior et al., 1998; Barca et al., 2006). The self-teaching hypothesis has been tested in different languages (Hebrew, English, and Dutch), with the findings suggesting that few exposures are required to form orthographic representations and pass from a sublexical to a lexical reading. However, this transition is more difficult for children with dyslexia (Manis, 1985; Reitsma, 1989). Additionally, it has been suggested that dyslexics are inefficient in learning graphemic materials because they were slower than controls in the learning rate of novel words when previous experience with texts was minimized (Pontillo et al., 2014).

Different methodologies have been proposed to investigate when the orthographic representations are formed (writing from dictation, choosing between several homophones of the target stimulus, reading latencies, etc.), but one widely recently used is based on the reduction of the length effect. From the study by Weekes (1997), it is well known that in typical readers, word length (number of letters) has a large influence on reading unfamiliar words and pseudowords, but has a small effect on low frequency words and no effect at all on high frequency words. The explanation, according to the dual route model, is that low frequency words and pseudowords are read in a serial or sublexical way, so the more graphemes, the larger the latencies. On the other hand, familiar words have a representation in the orthographic lexicon; so, when reading familiar words, all of the letters are identified in parallel, and the difference between the latencies of short and long words disappears (Coltheart et al., 2001). Therefore, the formation of orthographic representations will be reflected in a reduced length effect after repeated exposures to pseudowords (repeated reading). As a demonstration of this effect, Maloney et al. (2009) asked a group of participants to read the 100 Weeks study. They found that reading times were getting shorter, but more crucially, there was a reduction in the length effect: differences between long and short non-words became increasingly smaller.

In a recent study, Kwok and Ellis (2014) used this length effect methodology to study the formation of orthographic representations in dyslexic adults. Participants had to read a list of 24 non-words, half short and half long, repeated across ten blocks. Results showed a reduction in the difference in reading latencies between short and long words across blocks in normal readers, but dyslexics only showed convergence in the second session 7 days later. It seems that adult dyslexics need more exposures than control readers to create lexical representations.

With the same methodology, Suárez-Coalla et al. (2014) presented eight unfamiliar words, four long and four short, to a group of Spanish children with dyslexia and a control group to read in six different blocks. In a first experiment the unfamiliar words were presented within the context of a story and in a second experiment the words were presented in isolation. Reading and articulation times for the first and last block of the unfamiliar words were compared. In both experiments a decrease of the influence of length for the control group was found. However, for the dyslexic children, the influence of length remained unchanged after the repeated reading of the unfamiliar words. These results seem indicate that dyslexic children may be unable to develop orthographic representations, at least after six exposures, and that they may need to read each word more times.

Why do dyslexic children need more exposures to the words than normal children? It is quite possible that these results are a consequence of the difficulties they have in using grapheme–phoneme rules. Slow and inaccurate reading could prevent these children from developing orthographic representations. If so, the difficulties in forming orthographic representations will be higher for words associated with difficult rules, as for example those containing context-sensitive graphemes (Rastle and Coltheart, 1998; Rey and Schiller, 2005; Barca et al., 2007).

The Spanish orthographic system has 30 graphemes and is highly consistent; however, the pronunciation of “c” and “g”depends on the letter that follows (e.g., the letter “g” is pronounced as /γ/ when it is followed by “a,” “o,” “u”; but it is pronounced as /χ/ when followed by “e” and “i”). Therefore, the Spanish orthographic system is transparent, but contains some context-sensitive graphemes. Reading words and pseudowords is affected by graphemic complexity (complex GPC rules) in different languages (English: Rastle and Coltheart, 1998; French: Rey and Schiller, 2005; Italian: Barca et al., 2006). In Italian, a transparent language similar to Spanish, the graphemic complexity (contextuality) was tested in young Italian readers (third and fifth grades) using words with simple or contextual letter-sound conversion rules (Barca et al., 2007). In both groups, the words with contextual rules were read more slowly than words with simple rules. According to this result, we predict that it would be harder to build up orthographic representations for novel words that contain context-sensitive GPC rules than words that are made up of simple GPC rules only. We consider that this effect could be stronger for children with dyslexia than for normal readers because these rules are more difficult to learn and decode for dyslexics. So if these children have problems automating GPC rules, the context-sensitive GPC rules could entail an increased difficulty for them.

As a consequence, our goal in this study was to test, using the length effect reduction, if dyslexic Spanish children have problems in developing orthographic representations after repeated reading, and if these problems are greater when words include context-sensitive graphemes. Therefore, including context-sensitive graphemes would allow us testing whether dyslexics have poor learning of pseudowords because they have difficulties in learning and automating the grapheme–phoneme conversion rules.

In addressing that objective, we compared a group of Spanish dyslexic children with a group of normal readers on a task of repeated reading of pseudowords. Reading and articulation times were collected in order to discover if differences between short and long pseudowords decreased after repeated reading, and if this reduction of length effect was context-sensitive. In addition to reaction times (RTs), we have included articulation time (ATs), following previous studies (Davies et al., 2012; Suárez-Coalla and Cuetos, 2012), where ATs was a measure sensitive to the reader’s ability and characteristics of the stimuli.

## EXPERIMENT

### PARTICIPANTS

A total of 50 children took part in the study, all native Spanish speakers with normal, or corrected to normal vision and without any known cognitive impairment (apart from dyslexia). Children did not have sensory disorders. They all had received adequate schooling. Twenty five were dyslexics: their ages ranged between 8 and 13 years (*M* =10.36, SD = 1.5) and 25 were normal readers (*M* =10, SD = 1.5). Both groups were matched by gender (13 female and 12 male) and age. The dyslexic children were attending a private center for individualized treatment and received special attention in school. Both groups shared the same social background (middle-class families in all cases). For the diagnosis of the dyslexic children, in addition to the Wechsler Intelligence Scale for Children (WISC; Wechsler, 2001), a Spanish reading process assessment battery – PROLEC-R (Cuetos et al., 2007) was used. The battery was administered individually and required the child to read aloud a list of 40 words and pseudowords as quickly and as accurately as they could. These words varied quite broadly in frequency (high or low) as well as in length (five and eight letters). Accuracy and reading speed (measured as the time taken to complete the task) were scored. Children in the control group were also assessed using the PROLEC-R battery and the WISC test. The average intelligence quotient (IQ) in the dyslexic group was 106, ranging from 90 to 116; in the control group the mean IQ was 115 ranging from 95 to 126. Both groups were matched regarding performance IQ; the dyslexic children differed significantly from the control group in verbal IQ (*p* =0.006; common in people with dyslexia, and reported in other studies, e.g., Perea et al., 2014). Reading scores varied between the dyslexic and the control group; besides the dyslexic group scores were 1.5–2 SD below the average for each age category in the reading assessment battery (see **Table 1**). Furthermore, we confirmed significant differences between groups (dyslexics vs. controls) in reading accuracy of words [*t*(48) = -5.18; *p* < 0.001]; reading speed of words [*t*(48) = 4.90; *p* < 0.001]; reading accuracy of pseudowords [*t*(48) = -7.62; *p* < 0.001]; and reading speed of pseudowords [*t*(48) = 5.88; *p* < 0.001].

**Table 1:** Summary of participants’ characteristics.

Group	Dyslexics	Controls
	*M* (SD)	*M* (SD)
Age	10.36 (1.50)	10.00 (1.50)
IQ	106.45 (6.92)	115.38 (7.41)
- verbal IQ	102.2 (9.08)	114.7 (11.61)
- performance IQ	107.6 (7.80)	109.05 (12.01)
Word accuracy (out of 40)	35.08 (4.01)	39.34 (0.83)
Word speed in sec (out of 40)	79.70 (47.26)	30.78 (7.41)
Word/sec	0.72 (0.32)	1.43 (0.35)
Pseudoword accuracy (out of 40)	29.37 (4.40)	36.47 (2.33)
Pseudoword speed in sec (out of 40)	101.25 (38.38)	53.60 (13.89)
Pseudoword/sec	0.46 (0.11)	0.85 (0.17)

*IQ, intelligence quotient; M, mean; SD, standard deviation.*

The study was approved by the Ethics Committee of the Department of Psychology, University of Oviedo. Before performing the experiments, informed written consent from all parents and teachers was obtained. A document was given to the parents describing the objectives of the study, the type of tasks to be performed and their duration. The study involved only children whose parents signed the informed consent forms. Additionally, before starting the experiment, tasks were explained to the children and they were asked if they agreed to participate in the study. All children agreed to participate in the tasks.

### MATERIALS

Sixteen pseudowords in Spanish, half including consistent graphemes (d, t, m, or p), and half context dependent graphemes (g, j, z, or c), were used for this experiment. The pronunciations of contextual dependent graphemes varies according to the vowel that follows, as explained above. Half of the pseudowords were short (four letters, two syllables) and half long (six letters, three syllables); all had a consonant vocal (CV) syllabic structure (e.g., mepa, polato, zuge, gukato). According to the orthographic depth hypothesis, learning the alphabetic code is influenced by the orthographic consistency (Seymour et al., 2003); therefore, there would be more reading errors and lower reading fluency when stimuli are inconsistent, as opposed to when they are consistent. This would in turn cause difficulties in the formation of orthographic representations.

### PROCEDURE

The participants were asked to read aloud the pseudowords which were presented in random order within each of six blocks. For each trial, this sequence was followed: an asterisk was placed as a fixation point for 500 ms; this was followed by a blank screen for another 500 ms, and then the pseudoword appeared on the screen for another 3500 ms. A pilot study was conducted to determine timing of this sequence. We found that a shorter time was insufficient for children with dyslexia, as they did not have time to read the entire stimulus; without being able to read the stimulus, it would have been impossible for them to form representations. After each block, a pause was marked and participants pressed the space bar to continue. Before conducting the experiment, six practice trials were run in order to familiarize the children with the reading task. Stimuli were presented through the DMDX software (Forster and Forster, 2003) in a laptop computer (12^′′^) using a 24-point Arial font, colored black on a white background. Once the children were seated, the following instructions appeared on the screen: “Some invented words are going to appear in the screen; you must read them aloud as quickly as possible and without making any mistake.” The task was performed individually in a quiet room at the children’s school, or in the private center. The test lasted approximately 15 min. The children were not corrected if they misread pseudowords, thus trying to simulate the natural conditions of individual reading (self-teaching). Once the data were gathered, they were analyzed with the CheckVocal (Protopapas, 2007) software in order to obtain the correct responses, the reaction and articulation times.

### ANALYSIS

Using the SPSS 19 statistical package, a mixed between-within subjects analysis of variance was conducted. Group (2: dyslexics *vs*. controls) was the between-subjects factor; and block (2: first *vs*. sixth), stimulus type (2: consistent vs. context dependent graphemes) and length (2: short *vs*. long) the within-subjects factors.

Two dependent variables were considered: RTs (the time from the stimuli appearing on the computer screen until the child began to read) and ATs (the time children spent reading the stimuli). The AT has not been widely used in the literature, although there are some studies that have used this measure (Davies et al., 2012; Suárez-Coalla and Cuetos, 2012). This measure seems interesting, as far as children are concerned, because the length effect in ATs could be an indicator (other than RTs) of serial reading. A length effect in the ATs of dyslexic children was found, which was interpreted as absence of orthographic representations and thus sequential reading (Suárez-Coalla and Cuetos, 2012). We used only the correct responses for the RTs and ATs analyses; these responses are important in enabling us to discover if the lack of automatization of phoneme–grapheme rules is the problem concerning orthographic representations.

### RESULTS

A total of 4,800 responses were obtained from both groups, with 2,400 responses from each group. Considering the six blocks of stimuli, the dyslexic group committed a total of 465 errors (19.37%), and 73 non-responses (3.04%). Thirty (1.25%) responses were considered outliers (2 SDs above or below the mean). In contrast, the control group had a total of 207 errors (8.62%), three non-responses (0.12%), and 23 (0.96%) responses that were considered outliers. In the following analysis, only RTs and ATs to correct responses were used.

#### Reaction times

**FIGURE 1:**
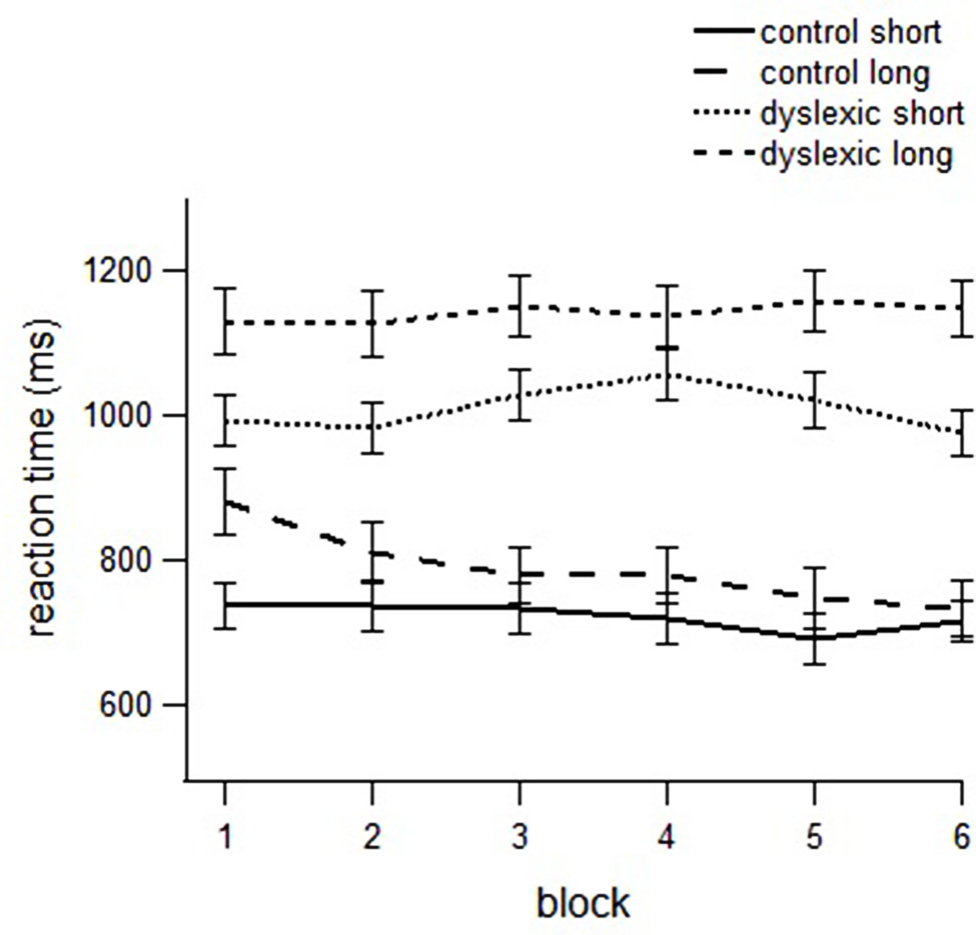
**Reading times to short and long pseudowords in dyslexics and controls across blocks**.

In the ANOVA we found a main effect of group [*F*(1,48) = 46.400, *p* < 0.001, partial μ^2^ = 0.502], with the dyslexic group slower than the control group; a block effect [*F*(1,48) = 4.690, *p* =0.036, partial μ^2^ = 0.093], as a consequence of the reduction in the RTs across blocks; a stimulus type effect [*F*(1,48) = 15.897, *p* < 0.001, partial μ^2^ = 0.257], with longer RTs for pseudowords with context-dependent graphemes than pseudowords with consistent graphemes; and a length effect [*F*(1,48) = 60.681, *p* < 0.001, partial μ^2^ = 0.569], due to RTs being faster for short than for long stimuli. We also found a block by group interaction [*F*(1,48) = 10.370, *p* =0.002, partial μ^2^ = 0.184], as the difference between RTs in first and last block was greater in the control than in the dyslexic group; a length by group interaction [*F*(1,48) = 6.727, *p* =0.013, partial μ^2^ = 0.128], showing a larger difference between short and long stimuli in the dyslexic than in the control group; and a block by length by group interaction [*F*(1,48) = 4.833, *p* = 0.033, partial μ^2^ = 0.095]. This latter interaction indicates that differences between short and long pseudowords decrease after repeated reading in the control group, but not in the dyslexic group (see **Figure 1**). In a more detailed analysis (comparing the first block with the rest of the blocks) it was found that the reduction of the length effect was only significant for the last block.

In order to further explore the results and confirm the decrease of length effect in the control group, RTs were separately analyzed for control and dyslexic children. In the analysis of the control group, we found a block effect, [*F*(1,24) = 17.927, *p* =0.001, partial μ^2^ = 0.428], indicating the decrease of RTs with the repeated reading in this group; a stimulus type effect, [*F*(1,24) = 18.051, *p* < 0.001, partial μ^2^ = 0.429], showing faster RTs in pseudowords with consistent graphemes than in pseudowords with context-dependent graphemes; and a length effect [*F*(1,24) = 23.211, *p* < 0.001, partial μ^2^ = 0.492], with longer RTs for long than short pseudowords.

We also found a stimulus type by length interaction [*F*(1,24) = 6.488, *p* = 0.018, partial μ^2^ = 0.213], as the length effect was smaller in pseudowords with consistent graphemes than in pseudowords with context-dependent graphemes; the block by stimulus type was close to significance [*F*(1,24) = 3.571, *p* = 0.071, partial μ^2^ = 0.130], indicating that RTs of pseudowords with consistent graphemes decreased more quickly with repeated reading; and a block by length interaction [*F*(1,24) = 18.629, *p* < 0.001, partial μ^2^ = 0.437], as the typical readers showed a length effect reduction with the repetitions.

By contrast, in the dyslexic group only a length effect was found [*F*(1,24) = 36.220, *p* < 0.001, partial μ^2^ = 0.622], indicating slower RTs for the longer pseudowords. The stimulus type effect was close to significance [*F*(1,24) = 3.776, *p* = 0.065, partial μ^2^ = 0.146], with longer RTs for pseudowords with context-dependent graphemes than for pseudowords with consistent graphemes. No effect of block was found indicating that reading times did not decrease after 6 exposures in the dyslexic group (see **Table 2** for the RTs in block 1 and block 6).

**Table 2:** Summary of RTs, ATs, and % of errors by dyslexics and controls, in blocks 1 and 6.

			Dyslexics			Controls		
Block	Stim. type	Length	RTs *M* (SD)	ATs *M* (SD)	% of errors	RTs *M* (SD)	ATs *M* (SD)	% of errors
B1	Consistent	Short	955 (182)	550 (248)	0.21	689 (114)	425 (73)	0.08
		Long	1103 (274)	884 (356)	0.79	846 (201)	621 (98)	0.25
	Cont. dep.	Short	1015 (268)	656 (170)	1.25	784 (180)	536 (90)	0.96
		Long	1177 (287)	977 (362)	1.33	911 (229)	717 (119)	0.71

B6	Consistent	Short	964 (199)	541 (261)	0.08	673 (122)	435 (76)	0.00
		Long	1148 (250)	820 (298)	0.67	724 (138)	587 (89)	0.04
	Cont. dep.	Short	992 (198)	624 (194)	1.29	760 (167)	526 (80)	0.78
		Long	1148 (272)	933 (363)	0.92	740 (153)	678 (98)	0.12

*Stim. type, stimulus type (consistent vs. context-dependent graphemes); Cont. dep., context dependent; B1, block 1; B6, block 6; RTs, reaction times; ATs, articulation times; *M*, mean; SD, standard deviation.*

#### Articulation times

**FIGURE 2:**
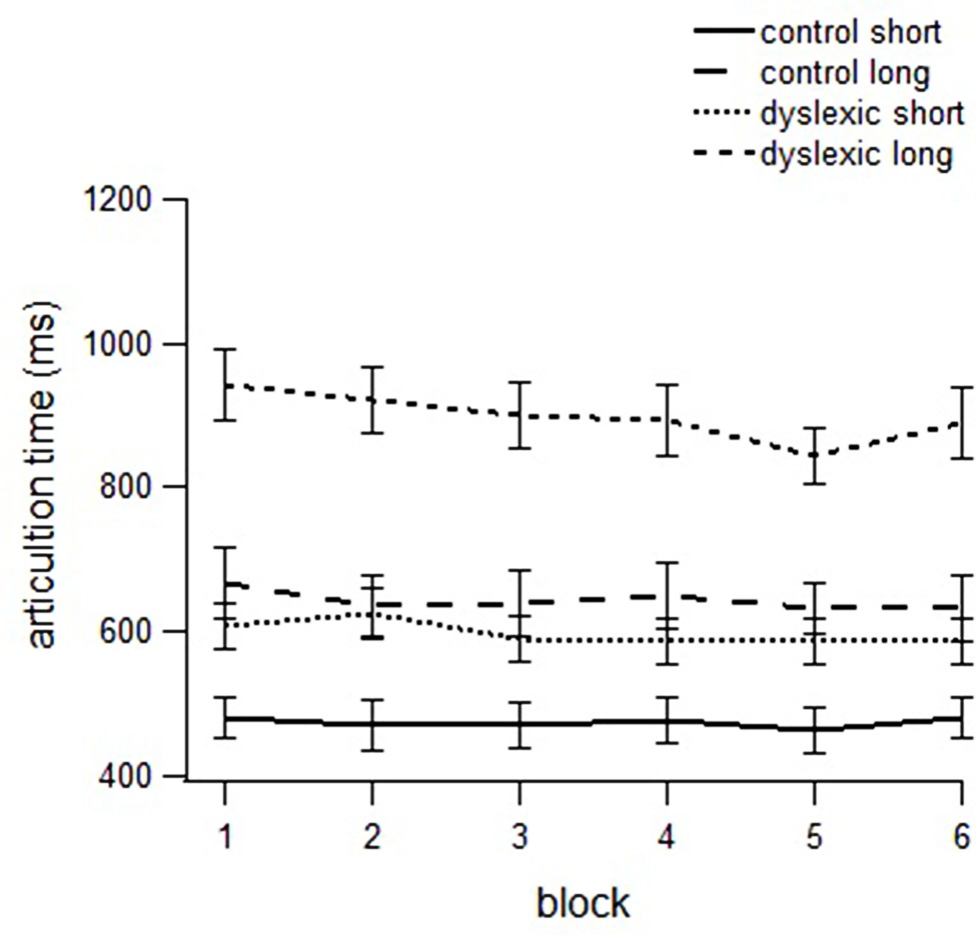
**Articulation times to short and long pseudowords in dyslexics and controls across blocks**.

In the ANOVA on articulation times, we found a main effect of group [*F*(1,48) = 13.166, *p* = 0.001, partial μ^2^ = 0.223], with longer ATs in the dyslexic group; block [*F*(1,48) = 6.267, *p* = 0.016, partial μ^2^ = 0.120], with longer ATs were longer in the first than in the sixth block; stimulus type [*F*(1,48) = 139.871, *p* < 0.001, partial μ^2^ = 0.753], as pseudowords with context-dependent graphemes took more time than pseudowords with consistent graphemes; length [*F*(1,48) = 356.407, *p* < 0.001, partial μ^2^ = 0.886], with shorter ATs for short than long pseudowords. Moreover, we found a length by group interaction [*F*(1,48) = 27.866, *p* < 0.001, partial μ^2^ = 0.377], and a block by length interaction [*F*(1,48) = 5.438, *p* = 0.024, partial μ^2^ = 0.106], indicating that the length effect was more evident in the dyslexic than in the control group and decreaded in the last compared to the first block. A more detailed analysis (comparing the first block with the rest of blocks) showed that the reduction of the length effect already appeared in block 5 (block by length interaction) and was maintained in block 6 (see **Figure 2**).

As with the RTs, we conducted separate analyses for controls and dyslexics. In the analysis of the control group data, we found a stimulus type effect, [*F*(1,24) = 278.345, *p* < 0.001, partial μ^2^ = 0.921], with faster ATs in pseudowords with consistent graphemes than in pseudowords with context-dependent graphemes; and a length effect [*F*(1,24) = 703.892, *p* < 0.001, partial μ^2^ = 0.967]. Moreover, the block by length interaction was significant [*F*(1,24) = 8.238, *p* = 0.008, partial μ^2^ = 0.256], as the length effect decreased across blocks.

In the group with dyslexia, we found a length effect, [*F*(1,24) = 144.292, *p* < 0.001, partial μ^2^ = 0.868], with shorter ATs for short than in long stimuli; and a stimulus type effect [*F*(1,24) = 45.494, *p* < 0.001, partial μ^2^ = 0.674], with longer ATs for pseudowords with context-dependent graphemes than pseudowords with consistent graphemes. By contrast, the block by length interaction was not significant, indicating that length continued to affect the ATs after six repetitions.

## DISCUSSION

In this study, we addressed the difficulty of Spanish-speaking dyslexic children in developing orthographic representations and investigated whether this difficulty is related to words that contain context-sensitive graphemes. In order to test this hypothesis, we compared children with dyslexia and typical readers using pseudowords with or without contextual grapheme–phoneme rules. The length effect reduction on reading speed, after repeated exposure, was considered as an indicator of orthographic representation development.

The results showed that dyslexic children were significantly slower at reading (RTs and ATs) than controls in all blocks, especially with long pseudowords. Additionally, children in the control group reduced the RTs across blocks (83 ms difference between the first and the sixth block), while the RTs of dyslexics remained the same through repetitions (2 ms difference between the first and the sixth block).

A critical finding was that typical readers showed a significant reduction of the length effect in the sixth block after repeated reading, i.e., the difference between short and long pseudowords was not significant in the last block (only 16 ms difference), suggesting development of orthographic representations. By contrast, dyslexic children continued to manifest a length effect in the sixth block (174 ms difference between short and long stimuli). These results are consistent with studies in other orthographic systems reporting that dyslexics have difficulties in storing the orthographic representations of words (Hogaboam and Perfetti, 1978; Manis, 1985; Reitsma, 1989; Ehri and Saltmarsh, 1995; Martens and de Jong, 2008; Kwok and Ellis, 2014).They also confirm results recently obtained with Spanish dyslexic children using the same methodology (Suárez-Coalla et al., 2014).

Regarding the orthographic consistency (i.e., consistent vs. context-dependent rules), we found that pseudowords with context-dependent rules are associated with longer RTs, ATs, and greater number of reading errors, in both dyslexics and controls. Therefore, it seems that context-dependent rules were more difficult to learn and automate, even for typical readers, in accordance with other studies (Rastle and Coltheart, 1998; Rey and Schiller, 2005; Barca et al., 2006). Nevertheless, the influence of the context-dependent graphemes on the formation of the orthographic representations seems to be stronger on the control group than on the dyslexic children, since in the normal children the reduction of the length effect after repeated reading was smaller for the pseudowords with these graphemes than for the pseudowords composed of consistent rules. On the other hand, in the dyslexic children, the length effect was similar for both types of graphemes. This suggests that dyslexics may be having problems forming orthographic representations even for words with consistent rules. In fact, dyslexic children were not able to develop orthographic representations with six exposures and continue using sublexical reading for all new words. They probably need more exposures to achieve a direct reading, as suggested by Kwok and Ellis (2014) in their study with dyslexic adults. Overall, we conclude that dyslexic children show a selective learning deficit in forming orthographic representations, independent of whether stimuli contained consistent or not context-sensitive rules. This independence from context-sensitive rules suggests a lexical locus for the learning difficulty of children with dyslexia.

Notably, dyslexic children remained slower than controls for both short and long stimuli. This highlights the known difficulties of dyslexics to read new words or pseudowords (Rack et al., 1992; Grainger et al., 2003; Suárez-Coalla and Cuetos, 2012). Their reading speed was more or less constant throughout the task, even showing longer RTs in the last block than controls on the first exposure. There is a possibility that inaccuracy interferes with orthographic learning because the correct mastering of the alphabetic code seems crucial; more times a word is accurately read, the greater the chances to store the representation in memory (Share, 1995). In this study, we found that dyslexic children made more errors than children without dyslexia, although an improvement in reading accuracy along the blocks occurred for both groups of children.

Besides RTs, dyslexics were also slower than controls in ATs. This measure, similarly to the RTs, decreased along the blocks and was affected by orthographic consistency and length, with more time needed to pronounce context-dependent and long pseudowords, than consistent and short ones. These results are in keeping with Davies et al. (2012) proposal that cognitive processes continue after response onset when word pronunciations is still not yet fully prepared. We should underscore, however, that the length effect was stronger in dyslexics and, furthermore, it did not decrease across the blocks, as it did for the controls. This means that dyslexic children continue doing a serial reading, even after several repetitions.

Finally, considering these results and those of other studies (Kwok and Ellis, 2014; Suárez-Coalla et al., 2014), it will certainly be interesting to perform a study with a larger number of repetitions, and in different days, in order to know if dyslexic children are able to develop orthographic representations with more exposures (Kwok and Ellis, 2014).

In summary, this study addressed the formation of orthographic representations in dyslexic children and the possible influence of context-sensitive rules. Previous studies have investigated this issue, but this is the first time the possibility that the formation of orthographic representations depends on the presence of context-dependent rules has been studied. Our results indicate that Spanish dyslexic children have problems to form orthographic representations (independent of the presence of context dependent graphemes) and continue using sublexical reading even after several exposures.

## Conflict of Interest Statement

The authors declare that the research was conducted in the absence of any commercial or financial relationships that could be construed as a potential conflict of interest.

## References

[B1] Barca L. Burani C. Di Filippo G. Zoccolotti P. (2006). Italian developmental dyslexic and proficient readers: where are the differences? *Brain Lang.* 98 347–351 10.1016/j.bandl.2006.05.001 16815542

[B2] Barca L. Ellis A. W. Burani C. (2007). Context-sensitive rules and word naming in Italian children. *Read. Writ.* 20 495–509 10.1007/s11145-006-9040-z

[B3] Coltheart M. Rastle K. Perry C. Langdon R. Ziegler J. (2001). DRC: a dual route cascaded model of visual word recognition and reading aloud. *Psychol. Rev.* 108 204–256 10.1037/0033-295X.108.1.204 11212628

[B4] Constantinidou M. Stainthorp R. (2009). Phonological awareness and reading speed deficits in reading disabled Greek-speaking children. *Educ. Psychol.* 29 171–186 10.1080/01443410802613483

[B5] Cuetos R. Rodríguez B. Ruano E. Arribas D. (2007). *PROLEC-R. Batería de Evaluación de los Procesos Lectores, Revisada. [Battery for the Assessment of Reading Processes-Revised]*. Madrid: TEA Ediciones.

[B6] Cunningham A. E. Perry K. E. Stanovich K. E. Share D. L. (2002). Orthographic learning during reading: examining the role of the self-teaching. *J. Exp. Child Psychol.* 82 185–199 10.1016/S0022-0965(02)00008-5 12093106

[B7] Davies R. Cuetos F. González-Seijas R. (2007). Reading development in a transparent orthography. *Ann. Dyslexia* 57 179–198 10.1007/s11881-007-0010-1 18041589

[B8] Davies R. Rodríguez-Ferrreiro J. Suárez P. Cuetos F. (2012). Lexical and sub-lexical effects on accuracy, reaction time and response duration: impaired and typical word and pseudoword reading in a transparent orthography. *Read. Writ.* 26 721–738 10.1007/s11145-012-9388-1

[B9] De Jong P. F. Bitter D. J. L. van Setten M. Marinus E. (2009). Does phonological recoding occur during silent reading, and is it necessary for orthographic learning? *J. Exp. Child Psychol.* 104 267–282 10.1016/j.jecp.2009.06.002 19608198

[B10] De Jong P. F. van der Leij A. (2002). Effects of phonological abilities and linguistic comprehension on the development of reading. *Sci. Stud. Read.* 6 51–77 10.1207/S1532799XSSR0601_03

[B11] Defior S. Justicia F. Martos F. (1998). Desarrollo del reconocimiento de palabras en lectores normales y retrasados en función de diferentes variables lingüísticas. *Infancia y Aprendizaje* 21 59–74 10.1174/021037098760403479

[B12] De Luca M. Barca L. Burani C. Zoccolotti P. (2008). The effect of word length and other sublexical, lexical and semantic variables on developmental reading deficits. *Cogn. Behav. Neurol.* 21 227–235 10.1097/WNN.0b013e318190d162 19057172

[B13] Ehri L. Saltmarsh J. (1995). Beginning readers outperform older disabled readers in learning to read words by sight. *Read. Writ.* 7 295–326 10.1007/BF03162082

[B14] Forster K. I. Forster J. C. (2003). DMDX: a windows display program with millisecond accuracy. *Behav. Res. Methods Instrum. Comput.* 35 116–124 10.3758/BF03195503 12723786

[B15] Grainger J. Bouttevin S. Truc C. Bastien M. Ziegler J. (2003). Word superiority, pseudoword superiority, and learning to read: a comparison of dyslexic and normal readers. *Brain and Lang.* 87 432–440 10.1016/S0093-934X(03)00145-7 14642545

[B16] Hatcher J. Snowling M. J. Griffiths Y. M. (2002). Cognitive assessment of dyslexic: students in higher education. *Br. J. Educ. Psychol.* 72 119–133 10.1348/000709902158801 11916468

[B17] Hogaboam T. W. Perfetti C. A. (1978). Reading skill and the role of verbal experience in decoding. *J. Educ. Psychol.* 70 717–729 10.1348/000709902158801

[B18] Kwok R. K. W. Ellis A. W. (2014). Visual word learning in adults with dyslexia. *Front. Hum. Neurosci.* 8:264 10.3389/fnhum.2014.00264 PMC4018562 24834044

[B19] Landerl K. (2001). Word recognition deficits in German: more evidence from a representative sample. *Dyslexia* 7 183–196 10.1002/dys.199 11881780

[B20] Landerl K. Wimmer H. Frith U. (1997). The impact of orthographic consistence on dyslexia: a German-English comparison. *Cognition* 63 315–334 10.1016/S0010-0277(97)00005-X 9265873

[B21] Maloney E. Risko E. F. O’Malley S. Besner D. (2009). Tracking the transition from sublexical to lexical processing: on the creation of orthographic and phonological lexical representations. *Q. J. Exp. Psychol.* 62 858–867 10.1080/17470210802578385 19107643

[B22] Manis F. R. (1985). Acquisition of word identification skills in normal and disabled readers. *J. Educ. Psychol.* 77 78–90 10.1080/17470210802578385

[B23] Martens V. E. G. de Jong P. F. (2008). Effects of repeated reading on the length effect in word and pseudoword reading. *J. Res. Read.* 31 40–54 10.1111/j.1467-9817.2007.00360.x

[B24] Nikolopoulos D. Goulandris N. Snowling J. (2003). “Developmental dyslexia in Greek,” in *Dyslexia in Different Languages* ed.Goulandris N. (London: Whurr).

[B25] Oney B. Goldman S. R. (1984). Recoding and comprehension skills in Turkish and English: effects of the regularity of grapheme–phoneme correspondences. *J. Educ. Psychol.* 76 447–568.

[B26] Perea M. Jiménez M. Suárez-Coalla P. Fernández N. Viña C. Cuetos F. (2014). Ability for voice recognition is a marker for dyslexia in children. *Exp. Psychol.* 24 1–8 10.1027/1618-3169/a000265 24962123

[B27] Pontillo M. De Luca M. Ellis A. W. Marinelli C. V. Spinelli D. Zoccolotti P. (2014). Failure to learn a new spatial format in children with developmental dyslexia. *Sci. Rep.* 4:4869 10.1038/srep04869 PMC4007079 24785494

[B28] Protopapas A. (2007). CheckVocal: a program to facilitate checking the accuracy and response time of vocal responses from DMDX. *Behav. Res. Methods* 39 859–862 10.3758/BF03192979 18183901

[B29] Rack J. P. Snowling M. J. Olson R. K. (1992). The nonword reading deficit in developmental dyslexia: a review. *R. Res. Q.* 27 29–53 10.2307/747832

[B30] Rastle K. Coltheart M. (1998). Whammies and double whammies: the length effect on nonword reading. *Psychon. Bull. Rev.* 5 277–282 10.2307/747832

[B31] Reitsma P. (1989). “Orthographic memory and learning to read,” in *Reading and Writing Disorders in Different Orthographic Systems* eds Aaron P. G. Joshi R. M. (New York: Kluwer) 31–44.

[B32] Rey A. Schiller N. O. (2005). Graphemic complexity and multiple print-to-sound associations in visual word recognition. *Mem. Cogn.* 33 76–85 10.3758/BF03195298 15915794

[B33] Seymour P. H. K. Aro M. Erskine J. M. (2003). Foundation literacy acquisition in European orthographies. *Br. J. Psychol.* 94 143–174 10.1348/000712603321661859 12803812

[B34] Share D. L. (1995). Phonological recoding and self-teaching: sine qua none of reading acquisition. *Cognition* 5 151–218 10.1016/0010-0277(94)00645-2 7789090

[B35] Share D. L. (1999). Phonological recoding and orthographic learning: a direct test of the self-teaching hypothesis. *J. Exp. Child Psychol.* 72 95–129 10.1006/jecp.1998.2481 9927525

[B36] Snowling M. J. (1995). Phonological processing and developmental dyslexia. *J. Res. Read.* 18 132–138 10.1111/j.1467-9817.1995.tb00079.x

[B37] Spinelli D. De Luca M. Di Filippo G. Mancini M. Martelli M. Zoccolotti P. (2005). Length effect in word naming in reading: role of reading experience and reading deficit in Italian readers. *Dev. Neuropsychol.* 27 217–235 10.1207/s15326942dn2702_2 15753047

[B38] Sprenger-Charolles L. Colé P. Serniclaes W. Lacert P. (2000). On subtypes of developmental dyslexia: evidence from processing time and accuracy scores. *Can. J. Exp. Psychol.* 54 88–104 10.1037/h0087332 10881393

[B39] Suárez-Coalla P. Cuetos F. (2012). Reading strategies in Spanish developmental dyslexics. *Ann. Dyslexia* 62 71–81 10.1007/s11881-011-0064-y 22215384

[B40] Suárez-Coalla P. Ramos S. Álvarez-Cañizo M. Cuetos F. (2014). Orthographic learning in dyslexic Spanish children. *Ann. Dyslexia* 64 166–181 10.1007/s1881-014-0092-5 25056668

[B41] Van den Broeck W. Geudens A. (2012). Old and new ways to study characteristics of reading disability: the case of the nonword-reading deficit. *Cogn. Psychol.* 65 414–456 10.1016/j.cogpsych.2012 22859020

[B42] Wechsler D. (2001). *Escala de Inteligencia para Niños-Revisión. [Intelligence Scale for Children-Revised]*. New York, NY: Psychological Corporation.

[B43] Weekes B. S. (1997). Differential effects of number of letters on word and nonword naming latency. *Q. J. Exp. Psychol. A* 50A, 439–456 10.1080/027249897392170

[B44] Wimmer H. (1993). Characteristics of developmental dyslexia in a regular writing system. *Appl. Psycholinguist.* 14 1–33 10.1017/S0142716400010122

[B45] Wimmer H. Goswami U. (1994). The influence of orthographic consistency on reading development: word recognition in English and German children. *Cognition* 51 91–103 10.1016/0010-0277(94)90010-8 8149718

[B46] Wimmer H. Schurz M. Strum D. Richlan F. Klackl J. Kronbichler M. (2010). A dual route perspective on poor reading in a regular orthography: an fMRI study. *Cortex* 46 1284–1298 10.1016/j.cortex.2010.06.004 20650450 PMC3073233

[B47] Yap R. L. Van der Leij A. (1993). Word processing in dyslexics: an automatic decoding deficit? *Read. Writ.* 5 261–279 10.1016/j.cortex.2010.06.004

[B48] Ziegler J. C. Perry C. Ma-Wyatt A. Ladner D. Schulte-Korne G. (2003). Developmental dyslexia in different languages: language-specific or universal? *J. Exp. Child Psychol.* 86 169–193 10.1016/S0022-0965(03)00139-5 14559203

[B49] Zoccolotti P. De Luca M. Di Pace E. Gasperini F. Judica A. Spinelli D. (2005). Word length effect in early reading and in developmental dyslexia. *Brain Lang.* 93 369–373 10.1016/j.bandl.2004.10.010 15862860

